# Isolated renal hydatid cyst: A rare presentation of echinococcosis

**DOI:** 10.1016/j.eucr.2025.103125

**Published:** 2025-07-16

**Authors:** Abel G. Wubie, Chernet T. Mengistie, Biruk T. Mengistie, Dereje G. Andargie, Daniel C. Teka, Temesgen H. Gebremeskel

**Affiliations:** a Assistant Professor of General Surgery, College of Medicine and Health Sciences, Bahir Dar University, Bahir Dar, Ethiopia; b School of Medicine, College of Health Sciences, Addis Ababa University, Addis Ababa, Ethiopia

**Keywords:** Cystic echinococcosis, Renal hydatid cyst, Nephron-sparing surgery, Scolicidal precautions

## Abstract

Isolated renal hydatid cysts are rare manifestations of cystic echinococcosis and often pose diagnostic and therapeutic challenges. We present a 40-year-old male with a large multivesicular cyst in the left kidney managed successfully with preoperative albendazole followed by nephron-sparing open cystectomy. Imaging revealed typical features, and histopathology confirmed Echinococcus granulosus. Surgical precautions minimized intraoperative spillage. This case highlights the importance of imaging in diagnosis and planning, and supports open nephron-sparing surgery with medical therapy as an effective approach for managing large renal hydatid cysts while preserving renal function.

## Highlights

• Renal hydatid disease is rare, with isolated cases presenting diagnostic challenges.
• Imaging revealed a complex cyst in the left kidney, confirming the diagnosis.
• The patient underwent successful nephron-sparing surgery with cyst excision.
• Early diagnosis and surgical intervention ensure favorable outcomes in renal hydatid disease.

## 1 Introduction

Hydatid disease (cystic echinococcosis) is a parasitic infection caused by the larval stage of Echinococcus granulosus, commonly found in pastoral regions worldwide.^1^ Endemic areas include parts of the Middle East, the Mediterranean basin, South America, Australia, and Central Asia.^1^ The liver (∼75 %) and lungs (∼15 %)are most frequently involved, while renal hydatid cysts are rare.^1^ Isolated renal involvement (without liver or lung cysts) accounts for only a small fraction of cases.^2^^,^^3^ Patients with renal echinococcosis may present with nonspecific symptoms such as flank pain, a palpable abdominal mass, or hydatiduria (passage of cyst material in urine).^3^^,^^4^ Diagnosis depends on imaging: ultrasound and CT typically show a well-defined multilocular cyst containing daughter cysts or internal septations. These imaging features form the basis of standardized classifications (Gharbi and WHO-IWGE) that stage cysts by complexity and viability.^4^ Serologic tests (e.g., ELISA) can support the diagnosis but have limited sensitivity and were not available in our setting. Surgical excision is the mainstay of treatment; nephron-sparing approaches are preferred if feasible to preserve renal function.^3^ Albendazole is administered preoperatively to inactivate the cyst and reduce the risk of anaphylaxis.^5^ We report a case of isolated renal hydatid cyst in a 40-year-old male and discuss its clinical presentation, imaging features (with radiologic classification), and surgical management. This case emphasizes important considerations for clinicians in endemic regions, including diagnosis and operative precautions required for this rare entity.

## 2 Case report

A 40-year-old previously healthy man presented with a three-year history of intermittent left flank pain that had recently intensified. He described the pain as a dull ache without radiation. He also noted intermittent left flank swelling and occasional darkening of urine, but no fever or weight loss. Physical examination revealed mild left flank tenderness but no palpable mass or organomegaly. Initial laboratory tests (CBC, serum chemistry including creatinine, urinalysis) were normal, with no eosinophilia. Serologic testing for Echinococcus was not available. Chest radiography was normal. He was a farmer living in a rural area, with frequent exposure to livestock and dogs.

**Fig. 1:**
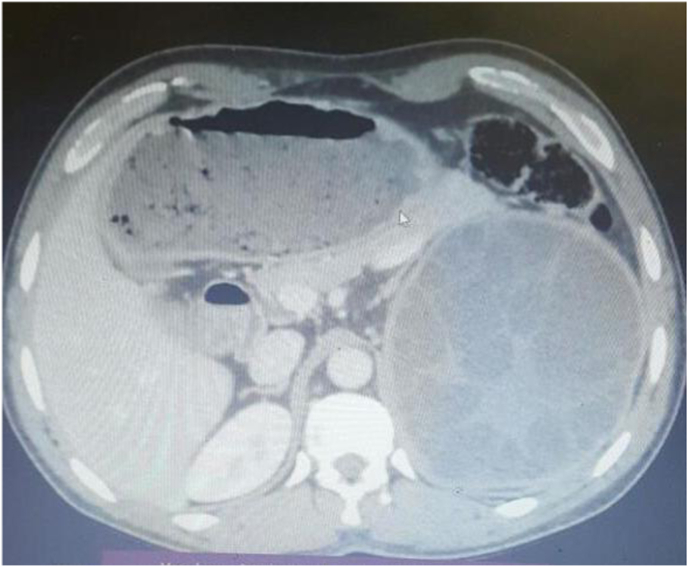
Pre-operative abdominal computed Tomography shows a mildly enhancing complex cystic mass with multiple peripheral small cysts and a central solid component situated in the superior part of the left kidney.

Abdominal ultrasound showed a large 12.3 × 8.6 cm multiloculated cystic mass at the upper pole of the left kidney with multiple septations. Contrast-enhanced CT confirmed a complex cystic lesion measuring approximately 12.4 × 12.0 × 11.0 cm (craniocaudal × transverse × anteroposterior) in the left upper pole (Fig. 1). The cyst contained numerous peripheral daughter cysts and a central soft-tissue component, displacing the spleen and tail of the pancreas. No hydronephrosis, hydroureter, or renal vessel invasion was seen. All other organs (liver, spleen, lungs) were unremarkable. In summary, imaging was consistent with a Gharbi type III multivesicular) hydatid cyst.

**Fig. 2:**
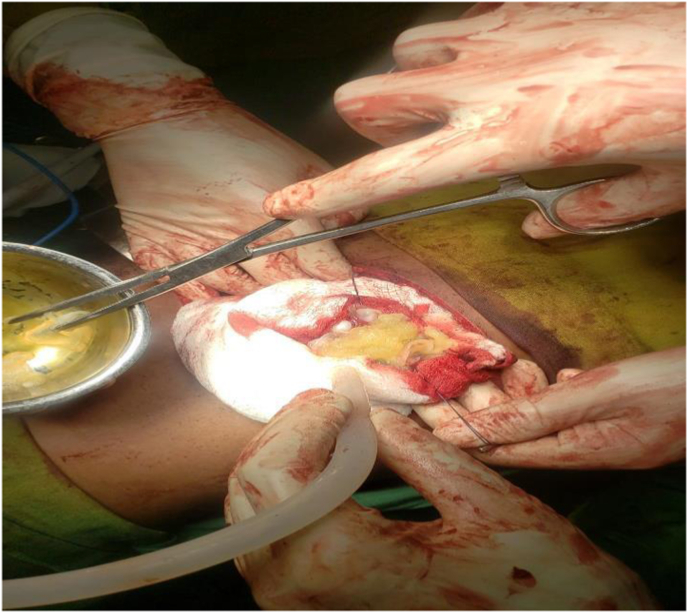
Intraoperative picture of cyst excision.

The patient received albendazole (400 mg twice daily) for one month. He then underwent open flank exploration and nephron-sparing resection of the renal cyst. Intraoperatively, the left kidney was exposed. Before opening the cyst, the operative field was surrounded with gauze pads soaked in 10 % povidone-iodine to act as a scolicidal barrier and to prevent spillage. The renal capsule was carefully incised over the cyst wall. Cyst fluid was aspirated in a controlled fashion to decompress the lesion; cytological examination of the aspirate revealed clear fluid with no acute inflammatory cells. After aspiration, hypertonic saline (20 %) was injected into the cyst cavity and left in place for 10 minutes as a scolicidal agent. The cyst was then gently opened, and daughter cysts and laminated membranes were removed en bloc (Fig. 2). The germinative and pericystic layers were dissected free from the renal parenchyma (partial pericystectomy), taking care to preserve cortical tissue. No obvious communication with the collecting system was found. The cavity was irrigated, and the defect in the kidney parenchyma was repaired with absorbable sutures. A drain was left in the retroperitoneum. Estimated blood loss was minimal. There were no intraoperative complications or hypotensive episodes.

**Fig. 3:**
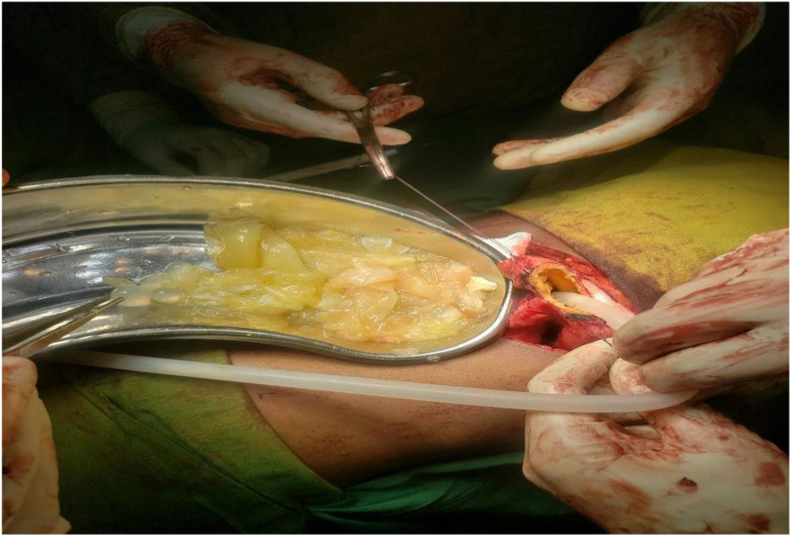
Intraoperative picture of a daughter cyst removal.

Gross examination of the excised cyst confirmed multiple daughter cysts and laminated membranes consistent with hydatid disease (Fig. 3). Histopathology of the specimen demonstrated the characteristic acellular laminated membrane and scolices of E. granulosus, confirming the diagnosis. He recovered well and was discharged on postoperative day 5. Follow-up ultrasound at 1 and 3 months showed normal renal architecture with no recurrence.

## 3 Discussion

Renal hydatid cysts are a rare manifestation of cystic echinococcosis. In general, hydatid disease most commonly affects the liver (∼75 % of cases) and lungs (∼15 %).^1^^,^^2^ Involvement of other organs such as the kidneys, spleen, heart, or central nervous system is unusual. The kidney is the third most common site, but still accounts for only about 2–4 % of cases.^3^^,^^4^ Isolated renal hydatidosis (with no concomitant hepatic or pulmonary cysts) is even rarer. A case series and literature reviews confirm that only a few dozen such cases are reported worldwide.^2^^,^^6^ In Ethiopia, reports of renal hydatid cysts are exceedingly scarce.

The pathophysiology involves the ingestion of Echinococcus eggs, hatching into oncospheres that travel through the bloodstream. Most become trapped in liver or lung capillaries; those that escape can lodge in systemic organs. For renal infection, it is thought that embryos pass through hepatic and pulmonary filters to reach the renal circulation.^7^^,^^8^ Once in the kidney, a single cyst usually forms in the cortex. Growth is slow (∼1 cm/year),^9^ so cysts can remain asymptomatic for many years. Eventually, symptoms such as flank discomfort, a palpable mass, or hematuria may prompt evaluation. Hydatiduria (passage of white, grape-like cyst material) is pathognomonic but occurs in only 10–20 % of renal cases^3^; it was absent in our patient.

Diagnosis relies primarily on imaging. Ultrasound is a useful first-line tool, often showing a complex cyst with daughter vesicles, septations, or floating membranes.^4^ CT and MRI provide more detail. In our patient, CT demonstrated a multivesicular cystic mass with peripheral daughter cysts and a thin capsule, without solid enhancement or adjacent invasion. These findings are characteristic of hydatid cysts.^4^^,^^8^ Based on such imaging, our lesion was classified as Gharbi Type III (WHO CE2) – a multilocular, multivesicular cyst.^8^

It is important to note that the differential diagnosis of a complex renal cyst includes simple cysts, multilocular cystic nephroma, cystic renal cell carcinoma, or abscess. In endemic areas, hydatid disease must be included in the differential even if exposures are not documented. Preoperative serology (ELISA for Echinococcus antibodies) can support the diagnosis, but these tests may not be available in many settings and can have false negatives, especially for solitary cysts.^3^^,^^5^ Indeed, serology was not performed for our patient due to resource constraints. Even when performed, a negative serology does not rule out hydatid disease.^5^

Therapeutically, our approach was guided by the lesion's size and location. In general, surgical excision is considered the gold standard treatment for renal hydatid cysts.^3^^,^^10^ Nephron-sparing surgery (cystectomy/pericystectomy) is preferred to preserve renal function if feasible.^11^ Total or partial nephrectomy is reserved for cases with extensive disease or a non-functioning kidney. Percutaneous PAIR (puncture–aspiration–injection–reaspiration) has been explored mostly for hepatic cysts; its role in renal cysts is limited due to the risk of spillage, anaphylaxis, and dissemination. According to guidelines, PAIR is best used for liver cysts of certain types and is contraindicated for superficial or multivesicular (“honeycomb”) cysts due to high spillage risk.^10^^,^^11^ Our patient's cyst was large, cortical, and multivesicular, so PAIR was not advisable. We opted for open cystectomy.

Intraoperatively, we took several precautions to prevent the spillage of protoscoleces. This included packing the operative field with gauze soaked in a scolicidal solution (10 % povidone-iodine).^6^ Similar techniques are routinely recommended; for example, Afghan case series describe isolating the hydatid cyst with povidone iodine soaked mops and aspirating cyst fluid before resection.^6^ After aspiration of the cyst fluid, we instilled hypertonic saline into the cavity for several minutes to kill any residual scolices. The cyst wall was then excised with partial removal of the pericyst, carefully sparing healthy renal tissue. In the literature, meticulous removal of the germinal layer is emphasized to prevent recurrence.^6^^,^^11^ No rupture or spillage occurred during our procedure. The decision to use an open approach (rather than laparoscopic) was based on the cyst's size and the need for maximum control; open surgery remains standard for large or complex hydatid cysts in many settings.^12^

Pre- and post-operative albendazole therapy was used based on standard practice. Albendazole (400 mg BID) for several weeks can sterilize the cyst, reduce intracystic pressure, and kill scolices, thereby minimizing recurrence.^5^^,^^11^^,^^13^ In resource-limited settings, monitoring for drug toxicity is important; our patient tolerated therapy well.

Our successful outcome (no complications, preserved renal function, no recurrence) is consistent with other reports that favor nephron-sparing surgery. The key teaching point is that even in endemic regions like Ethiopia, isolated renal hydatid cysts are often diagnosed late due to their rarity and non-specific presentation.^11^^,^^14^ Clinicians should maintain a high index of suspicion for hydatid disease when encountering complex renal cysts.^6^^,^^7^^,^^11^ Diagnostic limitations (unavailable ELISA, nonspecific labs) make imaging crucial.

## 4 Conclusion

Isolated renal hydatid cyst is a rare but important cause of renal cystic lesions, especially in endemic areas. This case illustrates the diagnostic challenges: nonspecific symptoms, normal basic labs, and the unavailability of serology. High-quality imaging (US/CT) was pivotal in suggesting the diagnosis. In settings with limited resources, relying on imaging patterns and epidemiology is essential. Treatment with nephron-sparing surgical excision combined with albendazole therapy can lead to excellent outcomes. Our experience confirms that even large renal hydatid cysts can be managed successfully with careful surgical technique (including scolicidal precautions) and preserve renal function. Increased awareness and timely intervention are key to managing this rare condition in Ethiopia and similar regions.

## CRediT authorship contribution statement

**Abel G. Wubie:** Writing – original draft, Supervision, Conceptualization. **Chernet T. Mengistie:** Writing – original draft, Resources, Data curation. **Biruk T. Mengistie:** Writing – review & editing, Writing – original draft, Visualization. **Dereje G. Andargie:** Writing – review & editing, Data curation. **Daniel C. Teka:** Writing – review & editing, Data curation. **Temesgen H. Gebremeskel:** Writing – review & editing, Visualization.

## Consent for publication

Written informed consent for publication of the case details was obtained from the patient.

## Ethics approval

IRB review and approval were waived for this case report.

## Funding

This research did not receive any specific grant from funding agencies in the public, commercial, or not-for-profit sectors.

## Declaration of interest

The authors declare that they have no known competing financial interests or personal relationships that could have appeared to influence the work reported in this paper.

## References

[1] Ishimitsu D.N., Saouaf R., Kallman C., Balzer B.L. (2010). Best cases from the AFIP: renal hydatid disease. Radiographics.

[2] Horchani A., Nouira Y., Kbaier I., Attyaoui F., Zribi A.S. (2000). Hydatid cyst of the kidney. A report of 147 controlled cases. Eur Urol.

[3] Zmerli S., Ayed M., Horchani A., Chami I., El Ouakdi M., Ben Slama M.R. (2001). Hydatid cyst of the kidney: diagnosis and treatment. World J Surg.

[4] Gupta S., Das C.J. (2022). Imaging of hydatid cyst of kidney, ureter, and urinary bladder. Br J Radiol.

[5] Centers for Disease Control and Prevention (CDC). Clinical Treatment of Echinococcosis [Internet]. Atlanta (GA): CDC; [updated 2019 Mar 27; cited 2025 Jun 22]. Available from: https://www.cdc.gov/echinococcosis/hcp/clinical-care/index.html.

[6] Misra A., Mandal S., Das M. (2021). Isolated renal hydatid disease: varied presentations, treatments, dilemmas, and the way ahead: case report series. Afr J Urol.

[7] Sachar S., Goyal S., Goyal S., Sangwan S. (2014). Uncommon locations and presentations of hydatid cyst. Ann Med Health Sci Res.

[8] El Amrani S., Imrani K., Moatassim Billah N., Nassar I. (2024).

[9] Singh V., Sinha R.J., Gupta D.K., Singh A., Pandey M., Bhat S. (2012). Isolated renal hydatid cyst: a report of 2 cases. UroToday Int J.

[10] Akhan O., Ustünsöz B., Somuncu I. (1998). Percutaneous renal hydatid cyst treatment: long-term results. Abdom Imaging.

[11] Yousofi Darani H., Jafari R. (2020). Renal echinococcosis; the parasite, host immune response, diagnosis and management. J Infect Dev Ctries.

[12] Vuruskan E., Ercil H., Anil H. (2022). Comparison of laparoscopic and open surgery in the treatment of renal hydatid cysts. J Laparoendosc Adv Surg Tech.

[13] Pandey U., Timilsina B.R., Acharya S., Paudel U., Kc S.R., Pradhan S. (2023). Renal hydatid cyst in a child managed with albendazole. J Nepal Health Res Counc.

[14] Teklay M., Gm, Tesfay T. (2019). Echinococcosis: the status of cystic hydatidosis in Ethiopia. Acta Sci Microbiol.

